# Multivariate Analysis of Open Field Exploration Identifies Latent Spatial and Social Behavioral Axes in Domestic Dogs

**DOI:** 10.3389/fnbeh.2020.00125

**Published:** 2020-07-17

**Authors:** Budhaditya Chowdhury, Moira van Staaden, Robert Huber

**Affiliations:** Department of Biological Sciences, J.P. Scott Center for Neuroscience, Mind and Behavior, Bowling Green State University, Bowling Green, OH, United States

**Keywords:** multivariate analysis, behavior, dog, GPS tracking, open field activity

## Abstract

Recent methodological advances in studying large scale animal movements have let researchers gather rich datasets from behaving animals. Often collected in small sample sizes due to logistical constraints, these datasets are however, ideal for multivariate explorations into behavioral complexity. In behavioral studies of domestic dogs, although automated data loggers have recently seen increasing use, a comprehensive framework to identify complex behavioral axes is lacking. Dog behavioral studies frequently rely on subjective ratings, despite demonstrable evidence that these are insufficient for identifying behavioral variables. Taking advantage of dogs’ innate running abilities and readily available GPS data loggers, we extracted latitude-longitude coordinates from running dogs in a large field setup. By extracting multiple variables from each logged coordinate, we generated a complex dataset from limited numbers of dog runs. Individual variables were successful in classifying aerobic competence, social awareness, and different exploratory patterns of dogs. Multivariate analyses identified latent features in movement patterns of dogs which were primarily comprised of two behavioral axes: spatial acuity and social awareness. Individual dogs were then behaviorally classified into independent clusters through unsupervised learning. Interestingly, even though field dogs clustered primarily with each other in varying degrees of energetic exploration and handler focus, some house pets displayed moderately high exploration abilities as well. We expect our proof of principle quantitative pipeline to provide a robust framework for behavioral classification, generating case-control clusters based solely on complex behavioral axes, and greatly benefiting genetic association studies of dog behavior.

## Introduction

Simple behaviors are often more than meets the eye, requiring multivariate and multi-level analysis to better understand function (Tinbergen, 1963). In behavioral studies of domestic dogs, however, the challenges arise not from a paucity of characterization, but from a surfeit of it. A focus of majority of early dog behavior studies have been on subjective rating scales – although new research has seen increased use of quantitative variables (Gerencsér et al., 2013; Huber, 2013; Correia-Caeiro et al., 2020). Most of these rating scales categorize dog behavior into “prosocial” and “reactive” categories. Broadly, tendency to approach and withdraw in novel situations (Plutchik, 1971) playfulness and activity (Hart and Miller, 1985), and personality (Svartberg and Forkman, 2002; Jones and Gosling, 2005) belong to the former group, while reactivity and immaturity (Bradshaw and Goodwin, 1999) and aggression (Cattell and Korth, 1973; Netto and Planta, 1997; van den Berg et al., 2003) are “reactive” features. Constructed in a framework where dog responses are often scored after provocations from humans, these characterizations unsurprisingly fall short in describing canine behaviors. In fact, a recent meta-analysis covering 25 years’ publications of dog behavioral evaluation found no sufficient evidence for these to be reliable in evaluating shelter dogs (Patronek et al., 2019) (however, the extent to which shelter dog behavior may overlap with pure breeds, should be kept in mind while evaluating these results). On the other hand, the difficulties in describing dog-dog/dog-human dyadic interactions are their presumed functions which may, or may not, have any biological basis. In fact, descriptions of self-handicapping (Bauer and Smuts, 2007) social mimicry (Palagi et al., 2018) attention getting (Horowitz, 2009), and dominance and submission (van Kerkhove, 2004; Bradshaw et al., 2009; Cordoni et al., 2016), might reflect projections by the human testers more so than emergent behaviors–as recent controversies over play bows attests (Byosiere et al., 2016).

Characterizing behaviors of today’s domestic dogs on their own merit is indeed challenging. Dogs are believed to have emerged at least 15,000 years ago from multiple origins (Wang et al., 2016) in close partnership with humans. Some forms of selection for increased working efficiency may have begun at least 4,000 years ago in the Middle East and North Africa (Serpell, 1996). Since then a continued and persistent selective regimen has been followed in creating and maintaining contemporary dog-breeds. Often derived from a small founding population and thus subjected to inbreeding on a small number of morpho-behavioral traits, dogs display enormous between-breed phenotypic diversity (Parker et al., 2004). From guarding livestock (Anatolian Shepherd dog), working in Western Alaskan conditions (Malamute), to sheep herding (Border Collie) and hunting (English Pointer), the behaviors of domestic dogs now varies extensively based on the nature of anthropocentric tasks for which they were selected. In contrast, a large number of recent dog breeds have also been primarily selected for suitability as house-pets, whose behavior must also factor in to fully understand the complete behavioral repertoire of domestic dogs. This calls for careful design of quantitative experiments that encapsulate general behavioral dispositions across dog breeds.

From ancient to the modern ones, a unifying behavioral feature of dogs is open field exploration – dogs of all breeds, as well as shelter and feral dogs show varying degrees of open field running. Be it house pets or field dogs used in upland game bird hunting, movement in an open field transcends breed boundaries. This energetic behavior also lends itself to characterization from a social standpoint. Ideally suited to extract multiple phenotypes, it also permits light to be shed on correlated responses hypothesized to be driven by domestication (Trut et al., 2009). Mostly studied for morphological traits, the correlated response in dogs have looked at quantitative traits that gets co-selected under selection. Through a robust quantification of behavioral traits, it might be possible to use similar approaches for behavioral traits as well. Recent years have seen the use of satellite telemetry for quantifying animal movement (Tomkiewicz et al., 2010; Hussey et al., 2015; Kays et al., 2015). In the case of dogs, satellite telemetry is in its infancy (Britt et al., 2011) along with other inertial sensors such as accelerometers and gyroscopes (Gerencsér et al., 2013). The latter study succeeded in automatically differentiating between canine locomotor activities (standing, sitting, galloping, etc.) through supervised training algorithms. To the best of our knowledge, a quantitative multivariate study aimed at identifying latent behavioral axes and constructing behaviorally meaningful clusters based on these – has yet to be carried out.

In the present study we used satellite telemetry to generate rich datasets of animal coordinates while they explored a large open field in the presence of their human companions. Multiple features were extracted per-coordinate, and multivariate analysis was carried out to explore spatial- and social relationships in movement patterns. The goal of this project was to: (i) identify socio-spatial variables and explore correlated responses in such features, (ii) assess to what extent *a-priori* classifications on group identities are reflected in these phenotypes, (iii) characterize latent features in this multivariate space, and (iv) use unsupervised learning on these features to group individual dogs into behaviorally meaningful clusters.

## Methods

### Animals and Field Site

**FIGURE 1:**
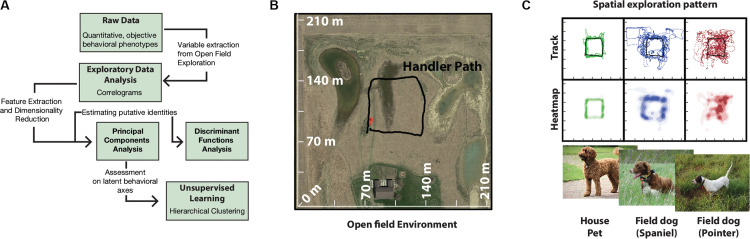
Quantitative analysis pipeline and behavioral setup. **(A)** Schematics of behavioral analysis. **(B)** Google Map image of the experimental field site with superimposed GPS tracks of human handler walking in an approximately square path. This path is representative of future handler trajectories during data collection. **(C)** Track and heatmaps of pilot exploration data of field usage by house, and field dogs. GPS track points were collected at 1 Hz, duration of total run per group was ∼40 min (*n* = 12) (Representative images, photo courtesy Bruce Williamson, Britta Mölders, BC).

A total of 16 dogs of different breeds were used (12 field dogs: 8 English Pointers, 4 Brittany Spaniels; 4 House pets: 2 Labradoodle, 2 Labrador Retrievers). All dogs were reported to be of similar sizes (over 40 lbs). For detailed description of how the dogs were distributed among handlers (see Supplementary Table 3). Owners escorted their dogs to the field site on the day of experiment where they were maintained in kennels (2.5 m × 2 m) and under conditions according to IACUC protocol (BGSU #08-018). A large field site (Figure 1B) near Tontogany, Ohio (41°24′36.2″N 83°46′24.7″W) provided for open field exploration. Field characteristics included even grass cover of ca. 0.5 m height, limited heterogeneity, and unobstructed wind flow. An approximate square path for the movement of the handler (Figure 1B) was marked by four corner stakes centered on the field.

### Experimental Procedure

We used a repeated measures design where each dog was tested three times. Inter-test durations were at least 30 min. For two of the three runs, live game birds were planted (in small bird cages, hidden in grass cover) to simulate upland hunting experience for field dogs. Birds were planted for runs of house pets as well, in order to maintain uniform stimulus. Dog collars were fitted with GPS receivers and handlers carried additional GPS receivers (Garmin Forerunner 205, weight 90 g). A single handler and one dog were permitted on the field site at any time. Dogs started their runs as the handler walked the designated path at a steady pace, with folded arms, and without issuing any commands or making eye contact with the dogs. Directionality of the handler-walk was randomly assigned prior to each run. A run was concluded when the dog found the hidden bird, or after a maximum duration of 10 min.

### Measurement of Phenotypes

Spatial data was collected from GPS receivers at 1 Hz frequency for both open field exploration of dogs and movement of handlers. Coordinates were transferred to a computer (Mac OS X 10.5.8), re-projected and plotted on Google Earth pro 5.0 for visualization. Once animal tracks passed quality control for artifacts and irregularities, the raw GPS data was Haversine transformed (geographic coordinate to metric measurements) for subsequent statistical analyses. Java DataGrinders^1^ was used to extract spatial and social variables from latitude-longitude coordinates of animal explorations. Linear variables were calculated across successive moves of GPS coordinates, and circular variables estimated using standard methods of circular statistics (Batschelet, 1981). To calculate means of circular variables, data were cosine transformed to avoid circular aggregation. Handler-dog variables were calculated for successive time-matched GPS coordinates. Scale invariant measurement (FractalD) was quantified using standard Mandelbrot method (Mandelbrot, 1967; Bovet and Benhamou, 1988). Descriptions of each variable are summarized in Table 1 (please consult Supplementary Figure 1 for detail).

**TABLE 1:** Description of quantified variables and estimation plots.

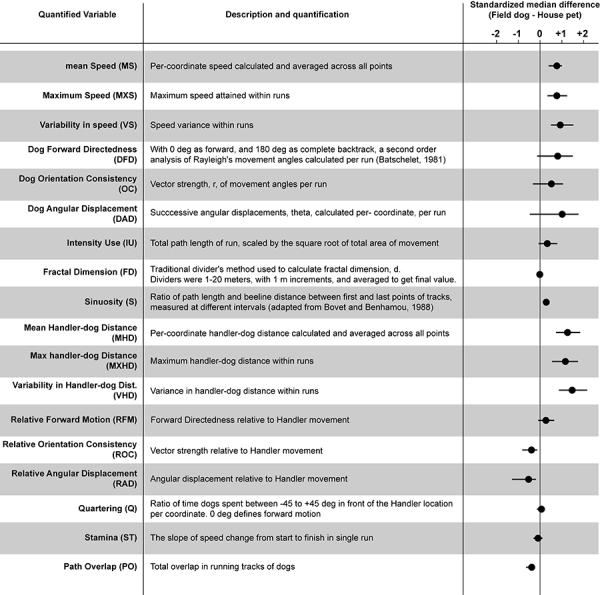

*Quantified variables are described with references (when appropriate). The median difference (field dog–house pet) for each variable was calculated on standardized data with median differences as dots, 95% confidence intervals are indicated by the ends of the bars. Confidence intervals were bias-corrected, and accelerated (Ho et al., 2019). Null hypotheses of zero difference was marked by the 0-vertical line.*

### Statistical Analysis

All behavioral data was standardized using log-transformation. Exploratory analysis of 18 variables was carried out with correlation analysis. Discriminant Function Analysis (DFA) was employed to identify features that distinguished dogs belonging to different groups. For DFA, field dog category was further subdivided into Pointers and Spaniels (pre-defined breed classifications) based on preliminary data of open field running (Figure 1C). Principal Components Analysis (PCA) was carried out for dimensionality reduction and identification of underlying behavioral axes. Hierarchical Cluster analysis (Ward’s method) on PCA axes was conducted for unbiased classification of dogs into behaviorally meaningful clusters (Cluster number was fixed at 4). For design of the data analysis pipeline see Figure 1A. All statistical analyses were carried out in JMP (JMP^®^, Version *13 Pro*. SAS Institute Inc., Cary, NC, 1989-2020). Estimation of Median differences were calculated using estimation plots analysis software (Ho et al., 2019).

## Results

### Deconstructed Open Field Exploration Identifies Correlated Behavioral Responses

**FIGURE 2:**
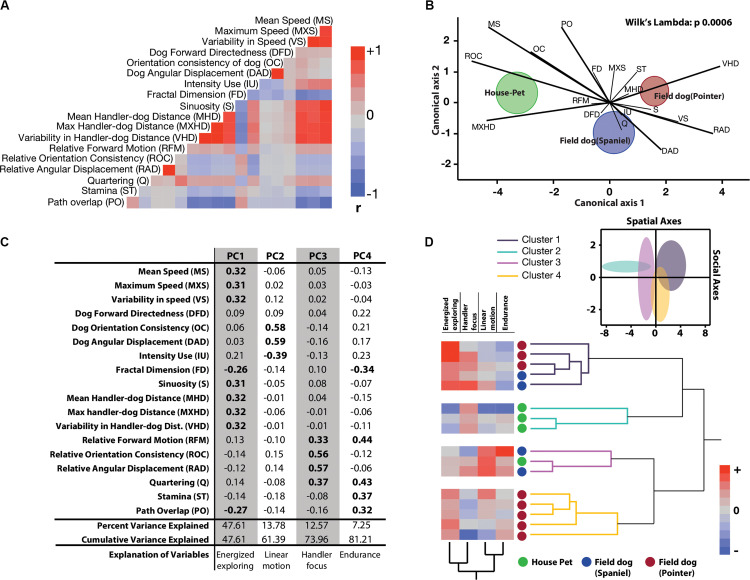
Quantitative characterization and identification of latent behavioral axes. **(A)** Correlation heatmaps of 18 linear and linearized angular variables show different degrees of collinearity and negative associations. Abridged letter codes are shown next to each variable. Each variable was generated from three repeated measures of dog runs. See this figure for descriptive ethograms. **(B)** Discriminant function analysis and biplot rays illustrate the extent and directionality of separation between three *a-priori* labeled groups of dogs (MANOVA, Wilks Lambda *p* = 0.0006). Canonical axis 1 and 2 represent linear combination of dependent variables as vectors. Biplot ray length and direction extending outward from a grand mean represent their ability to distinguish between labeled groups. Longer rays are more effective in separating labeled groups in their respective canonical axes. Circular clouds around each labeled group represent 95% confidence limits with a central multivariate mean. **(C)** Principal Components Analysis reduced the multidimensional dataset to four principal latent axes explaining 81% of variance observed in the total dataset. Based on the factor loadings of dependent measures on the four rotated principal components, salient behavioral identities were given for each axis. **(D)** Two-way hierarchical clustering of the first four principal component axes grouped 16 dogs in four behaviorally relevant clusters, and the behavioral axes in two clusters. Loadings of the latent axes is shown next to labeled dog-types along with the clustering dendrograms. Inset shows separation of four behavioral groups in scatterplot matrix of spatial (Energized exploring) and social (Handler focus) axes with 95% confidence ellipses.

We extracted 18 linear and circular variables from each open field exploration by dogs. None of these variables showed associations with age, field training, or sex of the animal (Supplementary Table 1). In broad categories of field and house dogs, the median difference values showed strong effect sizes in almost all of the univariate features (except Fractal dimension, Quartering, and stamina) (Table 1). Confidence intervals for each were calculated based on 5000 bootstrapped values. A correlogram (Figure 2A) revealed multiple correlated behavioral responses in open field running. As expected, speed variables (MS, MXS, VS) showed strong positive correlations with each other, and were in turn also strongly correlated with distance-to-handler variables (MHD, MXHD, VHD) (*ρ* > 0.87; *p* < 0.0001) (Figure 2A). Speed variables also showed strong negative correlations with FractalD (*ρ* >−0.61; *p* < 0.0001), stamina (*ρ* >−0.3; *p* < 0.02), and path overlap (*ρ* >−0.7; *p* < 0.0001). Orientation consistency correlated positively with angular displacement (tendency to move at a forward direction, 0 deg, cosine transformed as +1) (*ρ* > 0.98; *p* < 0.0001). FractalD of dogs’ running tracks were negatively correlated with sinuosity of running and handler relative distance features (*ρ* >−0.57; *p* < 0.0001). Finally, Path overlap of dogs’ runs were positively correlated with stamina of dogs (*ρ* = 0.38; *p* < 0.007). (For full correlogram on pearsons *ρ*, and corresponding *p*-values, see Supplementary Table 2).

### Conventional Categories of Dogs Display Salient Behavioral Features

Tracks and heat maps of open field exploration from pilot runs suggested marked visual differences between house pets, Spaniels and Pointers (Figure 1C). We therefore conducted discriminant function analysis to find behavioral variables segmenting these three labeled categories in multivariate space. The three categories differed significantly from each other based on 18 variables (MANOVA Wilk’s Lambda *p* = 0.0006). The canonical centroid plot distinguished the categories on the basis of aerobic competence (MS, VS, VHD), exploration strategy (PO, OC, DAD), and social features (MXHD, VHD, ROC, RAD) (Figure 2B). Field dogs showed clear separation from house pets on first canonical axes on measures of speed, orientation, and handler focus. The second canonical axis separated pointers and spaniels with measures of stamina, forward directedness, maximum speed attained, and quartering.

### Latent Spatial and Social Axes Cluster Dogs in Behaviorally Meaningful Groups

Discriminant function analysis suggested multiple behavioral features that separate labeled categories of dogs. In a subsequent dimensionality reduction step, Principal Components analysis was used to identify behavioral features with maximum variation, without any *a-priori* categorization. PCA produced 4 PC axes cumulatively accounting for over 81% of the total variance. Based on factor loadings (highlighted in bold) on the rotated principal component axes, they were labeled as Energized Exploring (PC1, percent variance explained = 47.61), Linear motion (PC2, percent variance explained = 13.78), Handler Focus (PC3, percent variance explained = 12.57), and Endurance (PC4, percent variance explained = 7.25) (Figure 2C). With reduced dimensionality of the multivariate space, a two-way hierarchical clustering algorithm was carried out with these four latent axes to assign a. dogs in behaviorally meaningful groups (without conventional labeling) and b. the behavioral axes into similar groups. As the clustering algorithm grouped dogs based on behavior alone, four major groups emerged. Field dogs (both Pointer and Spaniel) grouped together strongly in the first cluster, while house pets and pointers alone formed the second and fourth clusters, respectively. Interestingly the third group consisted of both house pets and field dogs. Spatial (Energized exploring) and social (Handler focus) axes were clustered together suggesting strong similarity. Heat maps of the behavioral axes explains these groups: strong exploratory drive in combination with social relationships (cluster 1), social focus alone (cluster 2), sustained linear motion with moderate social focus (cluster 3), and strong independent exploration with little to none human orientation (cluster 4) (Figure 2D). We visualized the separation of these clusters in the combined spatial-social behavioral space (Figure 2D, inset) with 95% confidence interval ellipses in a scatterplot. Cluster 1: cluster 2, cluster 1: cluster 3, and cluster 4: cluster 2 showed behavioral separation but other combinations showed considerable overlap. For separation of each cluster through serial behavioral axes as standardized cluster means (see Supplementary Figure 2).

## Discussion

Following a unique evolutionary history intertwined with humans, the majority of today’s domestic dogs show characteristic features. These are primarily based on form, with function often taking a back seat (Miklosi, 2016). Behavioral studies of dogs generally study differences between these pre-labeled breeds, using statistical inference that attempts to describe how observed data fit into pre-characterized groups. Relying primarily on observer or owner-based evaluations and questionnaires (Wiener and Haskell, 2016) conventional multivariate approaches are often unsuitable for behavioral traits (given the ordinal nature of data), and when applied with modifications (Svartberg, 2005) have had limited success. Efforts to understand behavior in groups of dogs in personality traits found no significant differences in working dogs (herding, guarding, and gun dog breeds) (Svartberg et al., 2005), suggesting insufficiency of behavioral characterization, overlap of behavioral phenotypes between labeled breeds, or possibly a combination of both.

In this paper we address this logjam in quantification by assessing innate dog behaviors with satellite telemetry, and exploring the extracted quantitative data with multivariate modeling, orthogonal transformations, and unsupervised clustering. Although most univariate measures studied suggested differences between working dogs and house pets (Table 1), the complex nature of the behavioral phenotypes could only be appreciated through multivariate analysis. Toward that goal we first assessed (via data visualization in multivariate space) if measured variables represented meaningful associations within each other, and with the small sample of dog types studied. The extracted measures showed correlations in independent and human-oriented features. This possibly aligns with the domestication hypothesis (Wheat et al., 2019) which posits multiple behavioral features to be correlated together as a result of selection for primary features. As a whole, pre-labeled categories of dogs showed strong signatures of separation in the multivariate space suggesting maintenance of functional traits in groups.

Secondly, and we believe the most important features of this proof of principle study is assigning meaningful attributes to linear combinations of rich behavioral quantification, that separated into spatial and social axes. Cumulatively explaining 60% of observed variation (PC Axes 1 and 3), these two behavioral axes most likely emphasize the nature of dog domestication history. These two axes also grouped together while assigning individual behavioral identities in two-way clustering. Grouped based on similarity of latent behavioral features, four behavioral clusters were imposed. Even though working dogs primarily grouped in their own clusters (although with high, and low social facilities), there were distinct signs of overlap in multivariate behavioral space. By representing this space in the spatial-social axes scatterplot, we provide quantitative measures for case-control analysis, and forward the possibility of carrying out genetic association studies. It is apparent from this study that assigning case-controls from breed identities alone can give rise to confusing results. For studies interested in identification of salient behavioral features between dog breeds, it should be kept in mind that larger sample sizes will provide better discriminating powers. Using the quantitative methodology forwarded in this paper, we anticipate the access to spatio-social feature quantification will greatly enhance such endeavors. Increasing sample sizes will also increase resolution of data for inferential statistical analysis, which we only briefly address in this paper.

In the rapidly advancing world of behavior genetics, the behavioral quantification aspect often plays catch up. As recent advances in automated animal tracking, machine learning, and deep learning algorithms begin closing this gap (Valletta et al., 2017), a quantitative behavioral methodology for studying domestic dogs is long overdue. Because of the limitations previously imposed, even with the access to the full genome sequence (Lindblad-Toh et al., 2005), the best understanding of dog behavioral genetics to date remains the early experiments carried out by Scott and Fuller (Scott and Fuller, 1965). With our proposed framework, we anticipate renewed efforts directed at understanding the genetic basis of complex social behavior in domestic dogs.

## Data Availability Statement

The datasets generated for this study are available on request to the corresponding author.

## Ethics Statement

The animal study was reviewed and approved by the IACUC protocol (BGSU #08-018). Written informed consent for participation was not obtained from the owners because oral consent was provided, following which, owners brought dog to experimental field.

## Author Contributions

BC, RH, and MvS designed the research and edited the manuscript. BC conducted the field experiments and wrote the manuscript. BC and RH analyzed the data. All authors contributed to the article and approved the submitted version.

## Conflict of Interest

The authors declare that the research was conducted in the absence of any commercial or financial relationships that could be construed as a potential conflict of interest.

## Footnotes

1 https://caspar.bgsu.edu/∼software/Java/JavaGrinders.html

## Supplementary Material

FIGURE S1 **(A)** Diagram depicting imaginary coordinates for handler (solid line) and dog (broken line) for 8 seconds (8 data capture each for handler and dog). Handler’s path shows consistent straight movement with a single 90 degree turn at move 6. The dog’s movement shows variability in running patterns. Calculation of mean handler-dog distance (a), mean handler-dog angle (b), orientation of dog (c), and sinuosity of path at every move (d/W; (W = W/2+W/2) were estimated from each trial. All other linear and circular measurements were calculated at each point and averaged over the entire run. **(B)** Schematics depicting High and Low loadings of important variables.

FIGURE S2 Separation of each behavioral cluster through serial Principal component axes measured in standardized cluster means.

TABLE S1 Coefficients of determination, *R*^2^, and associated *p*-values for individual behavioral variables associated with age, training, and sex of dogs.

TABLE S2 Pearson’s rho, and associated *p*-values in correlogram of quantified behavioral measures.

TABLE S3 Detailed description of dogs, and ownership among handlers. Training comprised mostly of holding pointing stance.
