# Supplementary figures and images for: Multivariate Analysis of Open Field Exploration Identifies Latent Spatial and Social Behavioral Axes in Domestic Dogs
Source: Front Behav Neurosci. 2020 Jul 17;14:125. doi: 10.3389/fnbeh.2020.00125 (PMC7380173; doi:10.3389/fnbeh.2020.00125)

**Speed**

**High**

**Low**

distance/unit time

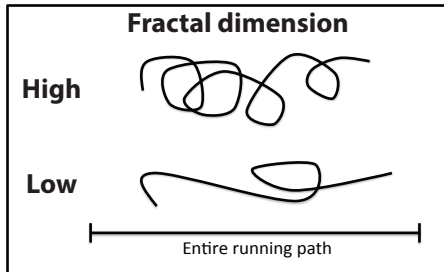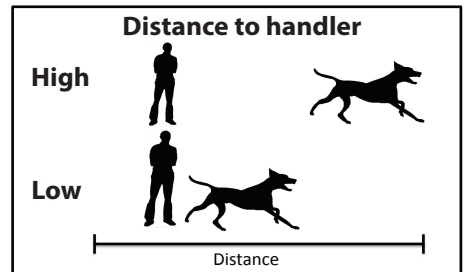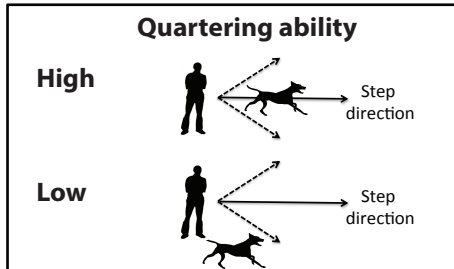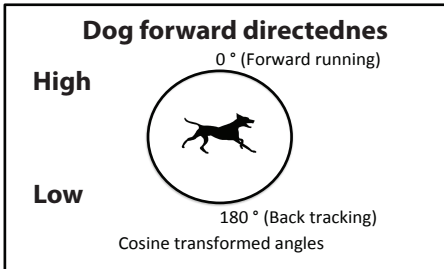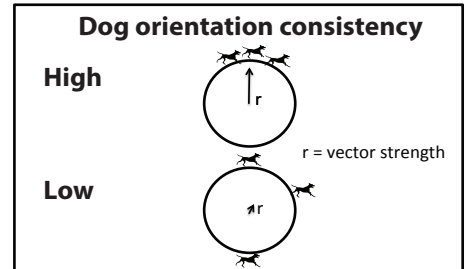

Supplement: FIGURE S1 — (A) Diagram depicting imaginary coordinates for handler (solid line) and dog (broken line) for 8 seconds (8 data capture each for handler and dog). Handler’s path shows consistent straight movement with a single 90 degree turn at move 6. The dog’s movement shows variability in running patterns. Calculation of mean handler-dog distance (a), mean handler-dog angle (b), orientation of dog (c), and sinuosity of path at every move (d/W; (W = W/2+W/2) were estimated from each trial. All other linear and circular measurements were calculated at each point and averaged over the entire run. (B) Schematics depicting High and Low loadings of important variables. [file Data_Sheet_1.PDF]

- Cluster 1
- Cluster 2
- Cluster 3
- Cluster 4

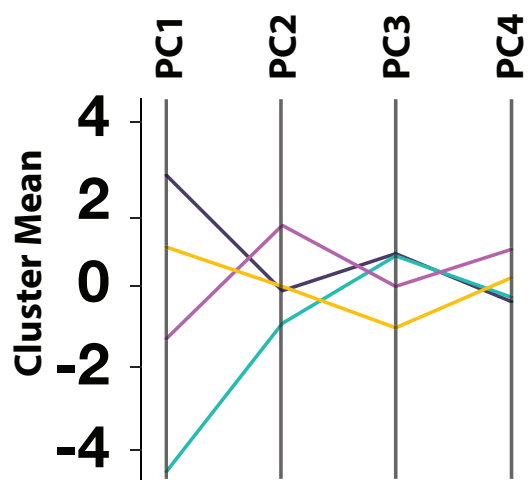

Supplement: FIGURE S2 — Separation of each behavioral cluster through serial Principal component axes measured in standardized cluster means. [file Data_Sheet_2.PDF]
