# Supplementary material for: Multivariate Analysis of Open Field Exploration Identifies Latent Spatial and Social Behavioral Axes in Domestic Dogs
Source: Front Behav Neurosci. 2020 Jul 17;14:125. doi: 10.3389/fnbeh.2020.00125 (PMC7380173; doi:10.3389/fnbeh.2020.00125)
Supplement: TABLE S1 — Coefficients of determination, R2, and associated p-values for individual behavioral variables associated with age, training, and sex of dogs. [file Data_Sheet_3.PDF]

Behavioral phenotype \ Predictor Variable	Age		Training		Sex	
	R^2	p-value	R^2	p-value	R^2	p-value
**Mean Speed**	0.06	0.32	0.001	0.89	0.02	0.59
**Maximum Speed**	0.1	0.18	0.0004	0.93	0.05	0.38
**Variability in speed**	0.05	0.36	0.001	0.88	0.12	0.18
**Dog Forward Directedness**	1e-5	0.98	0.13	0.16	0.04	0.45
**Dog Orientation Consistency**	0.15	0.13	0.23	0.06	0.1	0.22
**Dog Angular Displacement**	0.11	0.18	0.2	0.08	0.1	0.19
**Intensity Use**	0.06	0.35	0.002	0.84	0.04	0.42
**Fractal Dimension**	0.004	0.8	0.05	0.38	0.1	0.23
**Sinuosity**	0.01	0.6	0.07	0.31	0.07	0.32
**Mean Handler-dog Distance**	6e-4	0.92	0.15	0.13	0.03	0.47
**Max handler-dog Distance**	0.02	0.56	0.03	0.46	0.008	0.73
**Variability in Handler-dog Dist.**	3.9e-6	0.99	0.12	0.17	0.03	0.49
**Relative Forward Motion**	0.008	0.73	2.8e-5	0.98	0.07	0.29
**Relative Orientation Consistency**	0.04	0.41	0.02	0.53	0.05	0.84
**Relative Angular Displacement**	0.04	0.42	0.02	0.58	0.05	0.36
**Quartering**	0.13	0.16	0.01	0.61	0.005	0.78
**Stamina**	0.008	0.72	0.01	0.7	0.07	0.31
**Path Overlap**	0.01	0.69	0.04	0.42	0.02	0.53
