# Supplementary material for: Multivariate Analysis of Open Field Exploration Identifies Latent Spatial and Social Behavioral Axes in Domestic Dogs
Source: Front Behav Neurosci. 2020 Jul 17;14:125. doi: 10.3389/fnbeh.2020.00125 (PMC7380173; doi:10.3389/fnbeh.2020.00125)
Supplement: TABLE S3 — Detailed description of dogs, and ownership among handlers. Training comprised mostly of holding pointing stance. [file Data_Sheet_5.PDF]

Owner	Dog	Breed	Sex	Age	Training
Handler 1	Allie	Brittany Spaniel	F	2	0
	Maddie	Brittany Spaniel	F	2	0
Handler 2	Jake	Brittany Spaniel	M	4	2
	Ben	Brittany Spaniel	M	4	2
Handler 3	Browser	English Pointer	M	10	8
	Tank	English Pointer	M	5	3
	Thunder	English Pointer	M	4	2
Handler 4	Riley	English Pointer	M	3	1
Handler 5	Gradie	English Pointer	F	3	1
	Mickey	English Pointer	F	2	0
	Nugget	English Pointer	F	2	0
	Stormy	English Pointer	M	2	0
Handler 6	Zeus	Labrador Retriever	M	9	0
	Hera	Labrador Retriever	F	6	0
Handler 7	Rumi	Labradoodle	F	3	0
	Lola	Labradoodle	F	3	0
