# Supplementary material for: Multivariate Analysis of Open Field Exploration Identifies Latent Spatial and Social Behavioral Axes in Domestic Dogs
Source: Front Behav Neurosci. 2020 Jul 17;14:125. doi: 10.3389/fnbeh.2020.00125 (PMC7380173; doi:10.3389/fnbeh.2020.00125)
Supplement: TABLE S2 — Pearson’s rho, and associated p-values in correlogram of quantified behavioral measures. [file Data_Sheet_4.PDF]

Pearson's ρ \ p-value	Mean Speed	Maximum Speed	Variability in speed	Dog Forward Directedness	Dog Orientation Consistency	Dog Angular Displacement	Intensity Use	Fractal Dimension	Sinuosity	Mean Handler-dog Distance	Max handler-dog Distance	Variability in Handler-dog Dist.	Relative Forward Motion	Relative Orientation Consistency	Relative Angular Displacement	Quartering	Stamina	Path Overlap
**Mean Speed**		<.0001	<.0001	0.1545	0.8548	0.8560	<.0001	<.0001	<.0001	<.0001	<.0001	<.0001	0.0202	0.0281	0.0549	0.0135	0.0211	<.0001
**Maximum Speed**	0.87		<.0001	0.1470	0.2733	0.5445	<.0001	<.0001	<.0001	<.0001	<.0001	<.0001	0.0184	0.0216	0.0578	0.0037	0.0008	<.0001
**Variability in speed**	0.89	0.95		0.0915	0.0327	0.1065	0.0008	<.0001	<.0001	<.0001	<.0001	<.0001	0.0202	0.0279	0.0621	0.0073	0.0004	<.0001
**Dog Forward Directedness**	0.21	0.21	0.25		0.3726	0.3098	0.2892	0.1277	0.0620	0.1442	0.1550	0.1035	0.7988	0.8606	0.9202	0.2015	0.7679	0.2394
**Dog Orientation Consistency**	0.03	0.16	0.31	0.13		<.0001	0.0215	0.0015	0.8023	0.4808	0.6101	0.3866	0.7336	0.6715	0.7732	0.7784	0.2818	0.1667
**Dog Angular Displacement**	-0.03	0.09	0.24	0.15	0.98		0.0053	0.0185	0.9627	0.7984	0.9957	0.6915	0.4254	0.7766	0.8063	0.4379	0.4084	0.2426
**Intensity Use**	0.61	0.57	0.47	0.16	-0.33	-0.40		0.0003	<.0001	<.0001	<.0001	<.0001	0.0383	<.0001	0.0006	0.0236	0.6557	0.2529
**Fractal Dimension**	-0.61	-0.67	-0.73	-0.22	-0.45	-0.34	-0.50		<.0001	<.0001	<.0001	<.0001	0.0099	0.0062	0.0289	0.0171	0.1463	0.0027
**Sinuosity**	0.90	0.78	0.79	0.27	0.04	-0.01	0.55	-0.57		<.0001	<.0001	<.0001	0.0043	0.0603	0.1050	0.0128	0.0457	<.0001
**Mean Handler-dog Distance**	0.88	0.79	0.84	0.21	0.10	0.04	0.54	-0.64	0.84		<.0001	<.0001	0.0880	0.0578	0.0807	0.0171	0.0141	<.0001
**Max handler-dog Distance**	0.87	0.84	0.84	0.21	0.08	0.00	0.65	-0.69	0.83	0.95		<.0001	0.0407	0.0084	0.0161	0.0121	0.0360	<.0001
**Variability in Handler-dog Dist.**	0.88	0.83	0.86	0.24	0.13	0.06	0.57	-0.68	0.83	0.97	0.99		0.0774	0.0137	0.0220	0.0246	0.0131	<.0001
**Relative Forward Motion**	0.33	0.34	0.33	0.04	-0.05	-0.12	0.30	-0.37	0.41	0.25	0.30	0.26		0.6004	0.3498	<.0001	0.5160	0.1077
**Relative Orientation Consistency**	-0.32	-0.33	-0.32	-0.03	-0.06	-0.04	-0.54	0.39	-0.27	-0.28	-0.38	-0.35	0.08		<.0001	0.2994	0.8128	0.8339
**Relative Angular Displacement**	-0.28	-0.28	-0.27	-0.01	-0.04	-0.04	-0.48	0.32	-0.24	-0.25	-0.35	-0.33	0.14	0.99		0.1689	0.7952	0.8244
**Quartering**	0.35	0.41	0.38	0.19	-0.04	-0.11	0.33	-0.34	0.36	0.34	0.36	0.32	0.62	0.15	0.20		0.5091	0.1104
**Stamina**	-0.33	-0.47	-0.49	-0.04	-0.16	-0.12	0.07	0.21	-0.29	-0.35	-0.30	-0.36	-0.10	0.04	0.04	-0.10		0.0070
**Path Overlap**	-0.85	-0.70	-0.79	-0.17	-0.20	-0.17	-0.17	0.42	-0.79	-0.79	-0.73	-0.77	-0.24	0.03	0.03	-0.23	0.38	
